# Analysis of Solidarity Effect for Entropy, Pareto, and Gini Indices on Two-Class Society Using Kinetic Wealth Exchange Model

**DOI:** 10.3390/e22040386

**Published:** 2020-03-28

**Authors:** Gyuchang Lim, Seungsik Min

**Affiliations:** Department of Natural Science, Korea Naval Academy, Changwon 51704, Korea; econolim@daum.net

**Keywords:** kinetic wealth exchange model, stratified society, solidarity, Pareto exponent, Gini coefficient, entropy

## Abstract

It is well known that two different underlying dynamics lead to different patterns of income/wealth distribution such as the Boltzmann–Gibbs form for the lower end and the Pareto-like power-law form for the higher-end. The Boltzmann–Gibbs distribution is naturally derived from maximizing the entropy of random interactions among agents, whereas the Pareto distribution requires a rational approach of economics dependent on the wealth level. More interestingly, the Pareto regime is very dynamic, whereas the Boltzmann–Gibbs regime is stable over time. Also, there are some cases in which the distributions of income/wealth are bimodal or polymodal. In order to incorporate the dynamic aspects of the Pareto regime and the polymodal forms of income/wealth distribution into one stochastic model, we present a modified agent-based model based on classical kinetic wealth exchange models. First, we adopt a simple two-class society consisting of the rich and the poor where the agents in the same class engage in random exchanges while the agents in the different classes perform a wealth-dependent winner-takes-all trading. This modification leads the system to an extreme polarized society with preserving the Pareto exponent. Second, we incorporate a solidarity formation among agents belonging to the lower class in our model, in order to confront a super-rich agent. This modification leads the system to a drastic bimodal distribution of wealth with a varying Pareto exponent over varying the solidarity parameter, that is, the Pareto-regime becomes narrower and the Pareto exponent gets larger as the solidarity parameter increases. We argue that the solidarity formation is the key ingredient in the varying Pareto exponent and the polymodal distribution. Lastly, we take two approaches to evaluate the level of inequality of wealth such as Gini coefficients and the entropy measure. According to the numerical results, the increasing solidarity parameter leads to a decreasing Gini coefficient not linearly but nonlinearly, whereas the entropy measure is robust over varying solidarity parameters, implying that there is a trade-off between the intermediate party and the high end.

## 1. Introduction

Frequency distributions of a variety of statistics in social systems—such as income, wealth, city sizes, price-fluctuation of stock markets, and so on—show a power-law behavior. Among them, the distribution of income and wealth in a society was known to follow a power-law by V. Pareto in 1897 [1] and, since then, many empirical and numerical studies have been performed and newly discovered facts are reported over the whole range distribution functions [2,3,4,5,6,7,8]. The so-called Pareto law is valid only for the high rich people [9,10,11,12] and for the majority of non-rich people different distributions such as exponential and/or gamma-like functions are well fitted to the empirical statistics [13,14,15]. To explain the observed features of distributions of income and wealth, several agent-based models have been proposed on the basis of stochasticity and statistical mechanics. For the former, the variations of income and wealth are described in terms of stochastic terms, namely additive and multiplicative [6,16,17]. An additive term represents salaries while a multiplicative term represents a premium from investment proportionate to invested wealth. Their distribution is quite different from Gibbs and Gamma distributions and shows a power-law tail at large wealth and a sharp cutoff at small wealth. As for the latter, several physicists proposed kinetic exchange models of money or monetized wealth from analogy with an ideal gas in equilibrium for recent decades [7,8,18,19,20,21,22,23] although sociologist John Angle first proposed a class of stochastic processes for the universal emergence of inequality in wealth distribution [5]. By introducing saving propensities, gamma-like distributions were obtained as stationary distributions [24]. Kinetic exchange models with saving propensity shed a light on how rich people gain a huge wealth. A saving propensity is a key ingredient, which is correspondent to the multiplicative term in stochastic wealth models. When saving propensity is distributed heterogeneously to agents, a power-law tail appears in distribution functions as done by a multiplicative term in the stochastic wealth process [25]. 

For the kinetic exchange models, there have been many developments analytically and numerically [18,19,20,21,22,23]. Thus, we start our study from the existing kinetic exchange models, which are greatly adaptable for simulation and give the insights on empirical distributions of income and wealth in human societies. The goal of our study is to develop an agent based model incorporating the dynamic aspects of the Pareto regime [26] and the polymodal forms of income/wealth distribution [27,28] under the condition of constant total income/wealth. To this end, we first build a two-class society consisting of the lower end and the higher end, which are arbitrarily determined on 90–10 or 80–20 rules for simplicity. A two-class society is simply based on the fact of two-class structure of income distribution such as exponential bulk and power-law bulk [26]. Different from the random exchange rule taken in the classical kinetic exchange models [6,7,8], we adopt a wealth-dependent trading rule among agents belonging to different classes, according to which agents take part in a winner-take-all trade with their own wealth-weighted winning probability, respectively. By introducing a wealth-dependent trading rule, we simply replicate the unilateral flow of wealth via a premium from investment proportionate to invested wealth between the rich and the poor. The random exchange rules apply to agents belonging to the same class for both the poor and the rich. Then, we examine the change of wealth distributions over varying model parameters such as saving propensity and classifying criteria, that is, 90–10 and 80–20 rules. Second, we allow agents in the lower class to form solidarity against an agent in the upper class when they interact economically via wealth exchange. This additional restriction on the trading rule is motivated from activities of labor unions to a big as well as the government’s income inequality-mitigating policies such as increasing minimum wage and intensifying progressive taxes on people in the high-income brackets. By varying the solidarity parameter, we examine the change of income/wealth distributions and quantify the level of inequality in terms of inequality indices such as the Gini coefficient and the entropy measure. 

For comparative analyses, we apply the constraints such as a winner-take-all trading rule in a two-class society and the solidarity formation to the existing kinetic exchange models, which evolve to stationary wealth distributions after transient simulation time steps approximately to be 1000 Monte Carlo (MC) time. Then, we numerically investigate the impact of the additional constraints on the existing kinetic exchange models by examining the shape of wealth distribution, estimating the Pareto exponent and calculating the Gini coefficient, which is a standardized index for social inequality. Our simulation results will be given in Section 3.

The classical kinetic exchange models are classified as random exchange with no saving, homogeneous saving, and quenched heterogeneous saving [7]. Since a kinetic exchange model with heterogeneous saving propensity reproduces distributions very similar to empirical distributions of income and wealth, we perform a close comparative analysis on that model. For other two models, we give a graphical comparison. We also take two approaches, that is, a Lorenz curve-based Gini coefficient and a Shannon entropy in order to quantify the degree of inequality from model societies.

The paper is organized as follows. In Section 2 we give a brief description and a summary of features obtained analytically and numerically about those kinetic exchange models, and present our modified agent-based stochastic model. For the classical kinetic exchange models, we obtain the two-class classifying criteria numerically from their stationary distributions of income/wealth. All the simulation and numerical results are presented and discussed in Section 3. Concluding remarks are given in Section 4.

## 2. Kinetic Exchange Models 

In kinetic exchange models, two randomly chosen agents from a society consisting of N agents interact with each other through a pairwise exchange trading of a quantity x, referred to as monetized wealth. Agents are usually characterized by their current wealth $\left\{x_{i}\right\}$, i=1, 2, ⋯,N and by some parameters such as the saving propensity $\lambda_{i}$. The system evolves by the following microscopic dynamics

(1) $$x_{i}\left(t+1\right)=x_{i}\left(t\right)-△x,\;x_{j}\left(t+1\right)=x_{j}\left(t\right)+△x,$$

where △x is the money exchanged. It should be noticed that the total wealth of two agents is conserved during every transaction, $x_{i}\left(t+1\right)+x_{j}\left(t+1\right)=x_{i}\left(t\right)+x_{j}\left(t\right)$. Generally, the equilibrium or non-equilibrium distribution functions are determined by two ingredients of the microscopic dynamics, namely time-reversality of the exchange dynamics and boundary conditions on $\left\{x_{i}\right\}$. In the following, we briefly describe three kinetic exchange models and discuss how we modify them to fit the goal of our study. 

### 2.1. Kinetic Exchange Model without Saving

As described above, the quantity x represents the monetized wealth and △x is the money exchanged, which can have a constant value,

(2) $$△x=△x_{0}$$

or be a random contribution from both wealth

(3) $$△x=\epsilon x_{i}\left(t\right)-\left(1-\epsilon \right)x_{j}\left(t\right)$$

where ϵ is a random number uniformly distributed between 0 and 1, and is updated at every trade. The exchange rule in Equation (3) represents a random reshuffling of the wealth of two agents, since Equation (1) can be reformulated as

(4) $$x_{i}\left(t+1\right)=\epsilon (x_{i}\left(t\right)+x_{j}\left(t\right)),\;x_{j}\left(t+1\right)=\left(1-\epsilon \right)(x_{i}\left(t\right)+x_{j}\left(t\right)).$$

**Figure 1:**
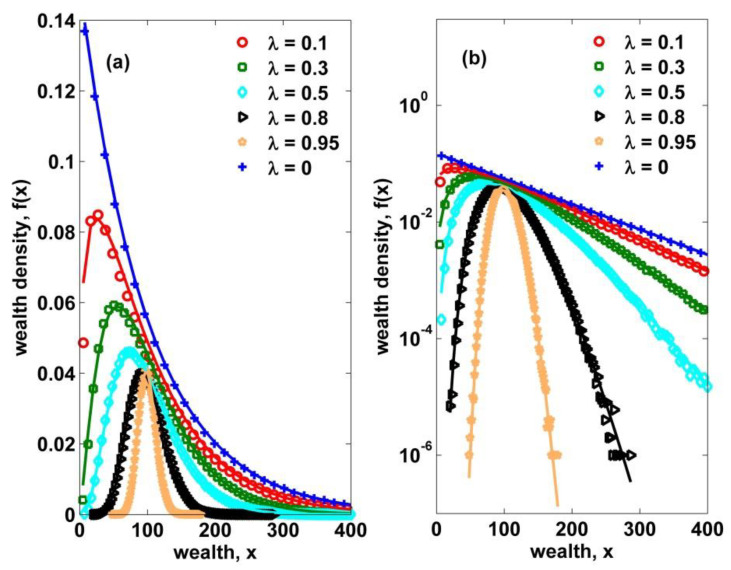
Linear (**a**) and semi-log (**b**) plots of the probability density of wealth from numerical simulations (dots) for homogeneous saving propensities with varying values in the interval [0,1), compared with the theoretical curves defined by Equations (8)–(10). When λ=0, the curve becomes the Boltzmann–Gibbs distribution. Also, when λ approaches 1, the peak shifts to the mean value and the fluctuations get smaller as predicted by the mode $x_{m}=3\lambda \langle x\rangle /\left(1+2\lambda \right)$ obtained from Equation (8).

Since these two dynamics are time-reversible, they, under the boundary condition of $x_{i}\left(t\right)>0$ and $x_{j}\left(t\right)>0$, lead to an equilibrium state characterized by the Boltzmann–Gibbs distribution [29],

(5) $$f\left(x\right)={\langle x\rangle }^{-1}\exp\left(-\frac{x}{\langle x\rangle }\right)$$

where the effective temperature $T_{\lambda }$ of the system is the average wealth; herein the homogeneous saving propensity λ=0, see Figure 1 where a perfect fitting with the theoretical curve is clearly observed. In fact, this exponential equilibrium distribution is theoretic-derived by maximizing the entropy of wealth distribution $S=-\int_{0}^{\infty }dxf\left(x\right)lnf\left(x\right)$ under the constraint of wealth conservation, using the method of Lagrange multipliers [30,31]. Although the above models seem to be too simple to describe the reality, there is a possibility that economic interactions among economic agents can be modeled in terms of simple statistical mechanics leading to universal statistical laws. 

### 2.2. Kinetic Exchange Model with Homogeneous Savings

A little more realistic economic exchange model, where all agents in the system are assigned a constant saving propensity, is presented. Here, all agents save a constant fraction λ of their own wealth before carrying out a pairwise random trade, and then exchange the remaining fraction (1−λ) of their wealth in the following way,

(6) $$x_{i}\left(t+1\right)=\lambda x_{i}\left(t\right)+\epsilon \left(1-\lambda \right)(x_{i}\left(t\right)+x_{j}\left(t\right)),\;x_{j}\left(t+1\right)=\lambda x_{j}\left(t\right)+\left(1-\epsilon \right)\left(1-\lambda \right)(x_{i}\left(t\right)+x_{j}\left(t\right)),$$

where △x in Equation (1) is given by

(7) $$△x=\left(1-\lambda \right)\left[\left(1-\epsilon \right)x_{i}\left(t\right)-\epsilon x_{j}\left(t\right)\right].$$

The corresponding equilibrium distribution is, by the numerical fitting, verified to be the gamma distribution which well fits the simulation results shown in Figure 1 [32],

(8) $$\left(\frac{\langle x\rangle }{n_{\lambda }}\right)f\left(x\right)\equiv f\left(\xi \right)=\frac{1}{\Gamma \left(n_{\lambda }\right)}\xi^{n_{\lambda }-1}e^{-\xi }=\gamma_{n_{\lambda }}\left(\xi \right)$$

where the wealth x is rescaled with respective to the effective temperature $T_{\lambda }$ as follows,

(9) $$\xi =\frac{x}{T_{\lambda }}$$

and the two parameters $n_{\lambda }$ and $D_{\lambda }$ are numerically estimated as follows

(10) $$n_{\lambda }\equiv \frac{D_{\lambda }}{2}=1+\frac{3\lambda }{1-\lambda }=\frac{1+2\lambda }{1-\lambda },\;T_{\lambda }=\frac{\langle x\rangle }{n_{\lambda }}=\frac{1-\lambda }{1+2\lambda }\langle x\rangle .$$

The parameter $D_{\lambda }$ plays the role of an effective dimension in an ideal gas system and the gamma distribution $\gamma_{n_{\lambda }}\left(\xi \right)$ is identical to the Maxwell–Boltzmann distribution of kinetic energy for a system of molecules at temperature $T_{\lambda }$ in $D_{\lambda }$ dimensions (only valid for integer or half-integer values of $n_{\lambda }$). Also, the mode of the gamma distribution f(x) of wealth is given as $x_{m}=3\lambda \langle x\rangle /\left(1+2\lambda \right)$ from Equation (8). 

Following this analogy between a closed economy model and an ideal gas kinetic theory, it should be noticed that an equipartition theorem leads to a relation between $\gamma_{n_{\lambda }}\left(\xi \right)$ and $D_{\lambda }$ [24],

(11) $$\langle x\rangle =\frac{1}{2}D_{\lambda }T_{\lambda }.$$

Thus, this equivalence is naturally extended to cases with real values λ≥0. 

Since λ varies between 0 and 1, the $D_{\lambda }$ increases from 2 to infinity. In a higher dimension, the fraction of exchanged kinetic energy between two colliding particles gets smaller and, at the same time, the effective temperature $T_{\lambda }$ decreases with increasing λ, indicating smaller fluctuations of x during exchange transactions as shown in Figure 1. One can notice that the mean amount of exchanged wealth in Equation (6) is given by (1−λ)〈x〉, which is approximately equal to $T_{\lambda }$, see Equation (10). 

### 2.3. Kinetic Exchange Model with Heterogeneous Savings

In fact, agents in the society are intrinsically heterogeneous in saving propensity. A further more realistic exchange model is established by assigning all the agents different saving propensities $\lambda_{i}$, which is distributed in the interval (0,1) [33,34]. The trading rule in this model is given as follows.

(12) $$x_{i}\left(t+1\right)=\lambda_{i}x_{i}\left(t\right)+\epsilon \left[\left(1-\lambda_{i}\right)x_{i}\left(t\right)+\left(1-\lambda_{j}\right)x_{j}\left(t\right)\right],\;x_{j}\left(t+1\right)=\lambda_{j}x_{j}\left(t\right)+\left(1-\epsilon \right)\left[\left(1-\lambda_{i}\right)x_{i}\left(t\right)+\left(1-\lambda_{j}\right)x_{j}\left(t\right)\right].$$

Or, equivalently, can be formulated through Equation (1) with △x given by

(13) $$△x=\left(1-\epsilon \right)\left(1-\lambda_{i}\right)x_{i}-\epsilon \left(1-\lambda_{j}\right)x_{j}.$$

**Figure 2:**
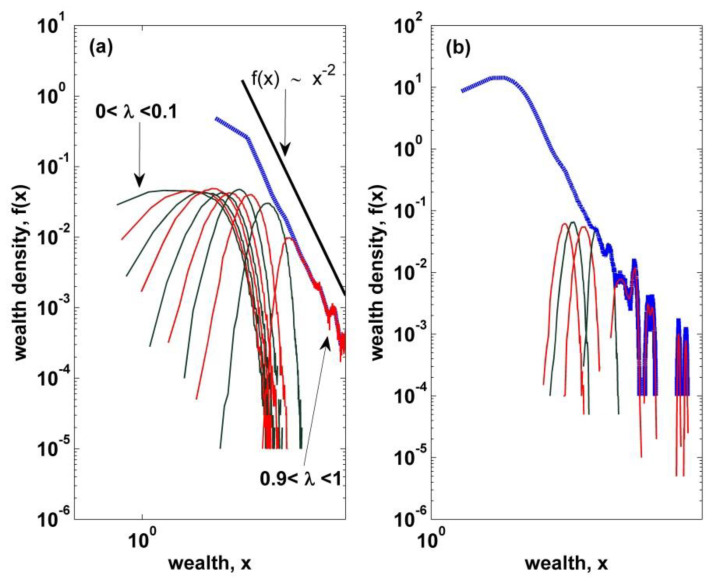
(**a**) The distribution of total wealth (blue dashed curve) is resolved into partial distributions (solid curves) corresponding to agents with saving propensities from the 10 intervals of width Δλ=0.1 of the λ range (0,1). (**b**) A partial distribution from the interval λ=(0.9,1.0) is further resolved into partial distributions from sub-intervals of width Δλ=0.02.

The most noticeable feature of this model, which is supported theoretically in several works, is that the stationary wealth distribution exhibits a robust power law at large vales of x,

(14) $$f\left(x\right)=x^{-1-\alpha }$$

with a Pareto exponent α=1 in the case of uniformly distributed λ and with α>1 if the density $g\left(\lambda \right)\sim {\left(1-\lambda \right)}^{\alpha -1}$ satisfying that g(λ)→0 for λ→1. As reported in [35], the wealth distribution of the single agents, belonging to a sub-interval of the λ range (0,1), is not of a power-law type but has a well-defined mode with an exponential tail, which is similar to the case with a constant saving propensity $\lambda_{0}$, as shown in Figure 2. Thus, the power law seems to arise from the superposition of these partial gamma-like distributions corresponding to the various batch of λ’s, where the average value is proportional to 1/(1−λ) and thus extended to very large values of x (see Figure 2). Roughly, the significant contribution from partial distributions to a power-law seems to be concentrated on the partial distributions with large saving propensities λ’s belonging to (0.9,1). 

### 2.4. Two-Class Kinetic Exchange Model with Wealth-Dependent Trading Rules

As reported from empirical studies of income distributions in USA and UK [13,14], there seems to be two distinct parts in a society, that is, the whole income distribution can be fitted by an exponential function in the lower part and a power-law function in the upper part. This fact reveals the existence of two-class structure in the American society and implies there are different mechanisms in wealth condensation for the poor and the rich, respectively. Based on these findings, we classify the society into two classes: the upper and the lower, respectively. Since it is unreasonable that a rich one and a poor one make a random exchange under the same condition, we impose additional restrictions on freely random exchange rules among agents as done in previous three representative kinetic models. If two agents belong to the same class, they can interact in the same manner with Equation (1). However, if they belong to different classes, they perform an exchange transaction on a random fraction of the lower agent’s wealth following the wealth-dependent trading rules defined in the below. That is, the exchange quantity △x is newly defined by

(15) $$△x=\varepsilon \times \min\left(x_{i}\left(t\right),x_{j}\left(t\right)\right)$$

where ε is a random variable uniformly distributed over [0,1] and is updated at every transaction. The random exchange rule is modified into a wealth-dependent trading with winning probabilities defined as follows,

(16) $$w_{i}\left(t\right)=x_{i}\left(t\right)/(x_{i}\left(t\right)+x_{j}\left(t\right))\;w_{j}\left(t\right)=x_{j}\left(t\right)/(x_{i}\left(t\right)+x_{j}\left(t\right))$$

where $w_{i}\left(t\right)$ and $w_{j}\left(t\right)$ are winning probabilities of agents, respectively, at the transaction time t. We applied the above rule to all three kinetic models with following classifying threshold values, which are arbitrarily determined based on 80–20 and/or 90–10 rules. The Table 1 contains the class-threshold values for three kinetic models with different saving parameters. 

### 2.5. Two-Class Kinetic Exchange Model with Solidarity

In reality, one-to-one trading between the agents belonging to the different classes is unrealistic when a rich agent is a company. Therefore, we consider a solidarity formation among the poor as done in activities by various labor unions. In our study, we assume that only agents in the lower class coalesce into big one union to perform the wealth-dependent trading with one agent in the upper class. In this case, the solidarity ratio α plays the role of critical control parameter. The whole trading procedures are summarized as follows:

1. Two agents are randomly selected.
2. Identify the class of each agent.
3. If they belong to the same class, then they perform the trading according to the rules described in Section 2.1 through Section 2.3, respectively.
4. If they belong to the different classes, the agent in the lower class gather partners according to the solidarity ratio η in the lower class and enter the wealth-weighted trade described in Section 2.4 with the following winning probabilities defined by
  (17) $$w_{i}\left(t\right)=\overset{N_{i}}{\underset{k=1}{\sum }}x_{k}/\left(\overset{N_{i}}{\underset{k=1}{\sum }}x_{k}+x_{j}\right)\;w_{j}\left(t\right)=x_{j}/\left(\overset{N_{i}}{\underset{k=1}{\sum }}x_{k}+x_{j}\right)$$
  where $N_{i}=\eta N_{lower}$+1, the $N_{lower}$ denotes the number of agents in the lower class.
5. If the agent i wins the trading, Δx is equally distributed to all the partners.
6. When the agent i loses the trading, only the agent i loses his own wealth with other partners preserving their own’s.

## 3. Numerical Results and Inequality Index

### 3.1. Wealth-Dependent Trading Rules Effect on Wealth Distribution of a Stratified Society

**Figure 3:**
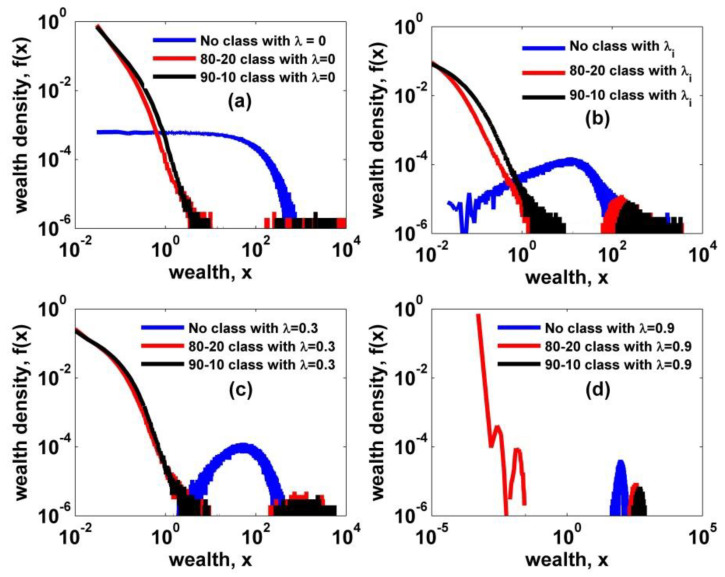
Wealth density functions of no-class and two-class cases are plotted for comparison. (**a**) A simple exchange model with no saving; (**b**) an exchange model with heterogeneous saving; and exchange models with homogeneous saving, (**c**) λ = 0.3 and (**d**) λ = 0.9. One salient feature is that a power-law in the lower part is clearly observed and the other exciting finding is that the differentiation is intensified with increasing homogenous saving propensity, as shown in (**c**) and (**d**).

In our study, we determine the classification threshold values of wealth estimated by rule of thumb based on the 80–20 and/or 90–10 rules for the representative three kinetic models, respectively, as summarized in Table 1. We start a simulation of each kinetic model for a society consisting of 1000 agents with 100 initial wealth each. In order to obtain the steady-state distributions for each simulation, we perform $10^{3}$ Monte Carlo (MC) time steps, where one MC time step is defined as 1000 times random exchanges among 1000 agents. Thus, during one MC time step, each agent can trade at least twice on average. Also, we adopt the ensemble method in order to ensure good statistics, which implies a good quality of wealth histogram. In a two-class effect simulation, we created 1000 ensembles for each condition. We presented the simulation results in Figure 3. By imposing the class boundary and the wealth-dependent winning probability rule on every trading among agents, we can observe that an extreme differentiation emerges with a perfect collapse of the intermediate part. In addition, we find two interesting facts: one is the power-law patterns observed in the lower part under the conditions of no saving, lower saving, and heterogeneous saving, and the other is the intensified differentiation with increasing homogeneous saving propensity. As for the latter case, an unclassified society with a high saving propensity is very close to an egalitarian society with very low Gini coefficient, numerically estimated to be 0.11 on ensemble average. However, about 80% of the total population falls into the extreme poverty by incorporating the classifying effect. This finding strongly supports that the wealth-dependent trading rule in a society can lead a normal or a near-equality society to extremely skewed society with an extreme poverty class. 

### 3.2. Solidarity Effect on Wealth Distribution of a Stratified Society

**Figure 4:**
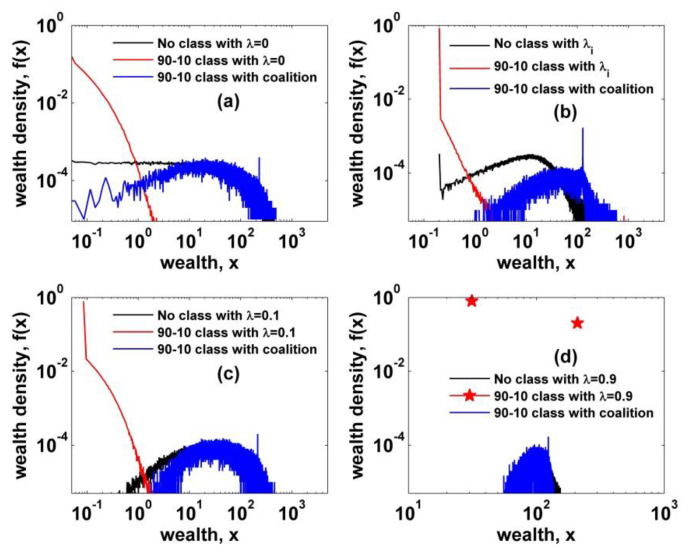
Wealth density functions of no class and two kinds of two-class cases are plotted for comparison. (**a**) An intermediate part is intensified by the solidarity effect while the lower and the upper parts shrink; (**b**) a similar behavior is observed and the peak indicates the class-threshold value; (**c**) and (**d**) also show the behavior of mitigating inequality by the solidarity. All solidarity parameters are set to η=0.1.

**Figure 5:**
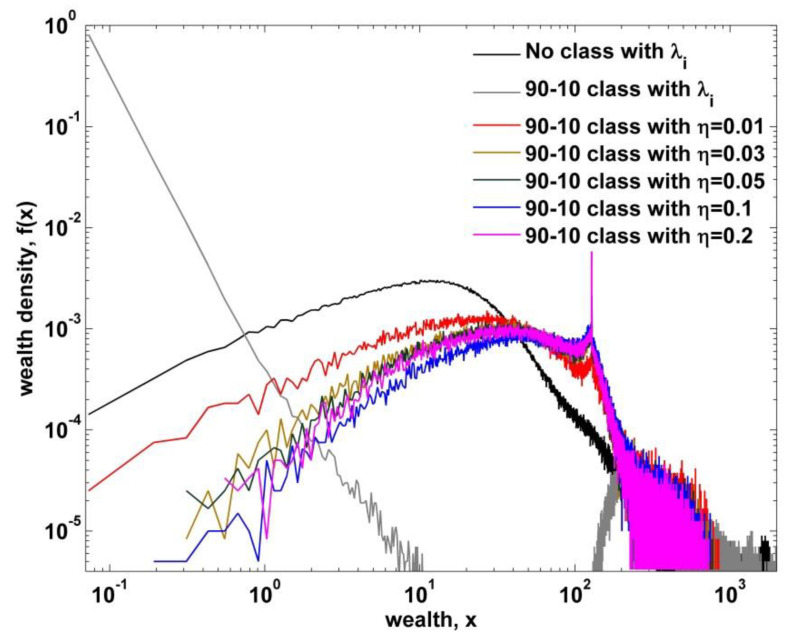
Wealth density functions plotted for the kinetic exchange models with heterogeneous savings of no class and two-class societies with varying solidarity parameters. There is no clear distinction between η=0.1 and η=0.2.

As shown in previous section, a two-class society with wealth-dependent trading rules leads the system into the perfect collapse of the intermediate part. Despite of the random exchange in each class, the unilateral flow of wealth from the poor to the rich completely polarizes society. Therefore, some countermeasures, such as activity of a labor union and/or government policies, must be taken to mitigate the social polarization developed in the above models. We allow agents in the lower class to form solidarity against the agent in the upper class in a wealth-dependent trade. Figure 4 shows the comparative numerical results for four cases with different conditions and parameters. Herein, we only consider those cases with two-class society based on 90–10 rule, since there is no clear difference between 80–20 and 90–10. Noticeably, we observe that there is a clear mitigating behavior of wealth inequality by the solidarity. As confirmed numerically in Section 3.1, a two-class society with wealth-dependent trading rules develops the society into extreme social polarization. However, just by introducing a solidarity condition to agents in the lower class, the society develops into more equalized stationary state. We also examine the behavior of inequality mitigation over varying solidarity parameter. To this end, we consider only the two-class society with heterogeneous saving propensity, which is much closer to reality. As shown in Figure 5, there is an apparent behavior of decreasing fraction of the poor as the solidarity parameter increases to η=0.1, over which no clear behavior of decreasing inequality is observed.

**Figure 6:**
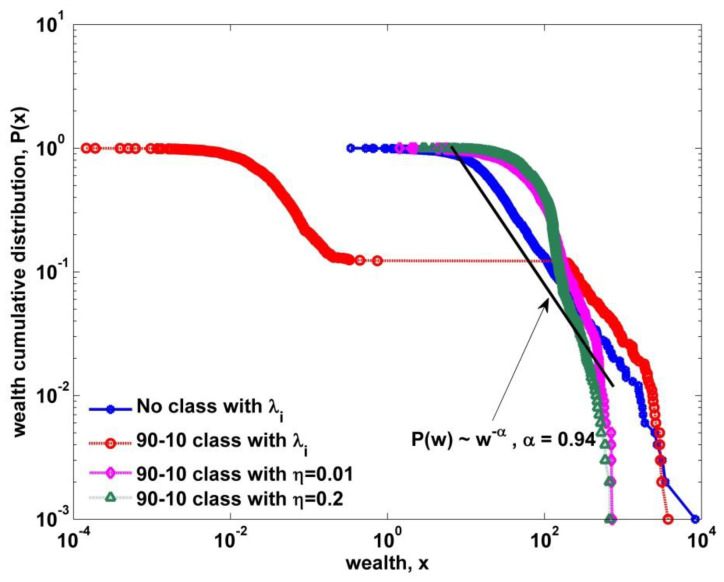
Wealth cumulative distributions plotted for the kinetic exchange models with heterogeneous savings of no class and two-class societies with varying solidarity parameters. For the no-class case, there is a large Pareto range covering more than 50% population. For the two-class with no solidarity, the high end still has the Pareto regime with a Pareto exponent of 0.94 and shows the clear collapse of the intermediate part. For the rest two cases, the Pareto regime shrinks too much and the descent gets steeper with a larger Pareto exponent while the intermediate part gets intensified.

However, it is unclear if there is a Pareto-regime in the upper class from Figure 5. Therefore, we examine the cumulative wealth distribution as shown in Figure 6, where we discovered two important behaviors. One is the robustness of the Pareto-law behavior in the high end. Even in the extremely polarized case, the Pareto exponent is almost invariant. The other is the deviation of Pareto exponent from being α=1. As the solidarity parameter gets higher, the Pareto regime gradually shrinks with increasing Pareto exponent. 

### 3.3. Lorenz Curve and Gini Coefficient as an Inequality Index

**Figure 7:**
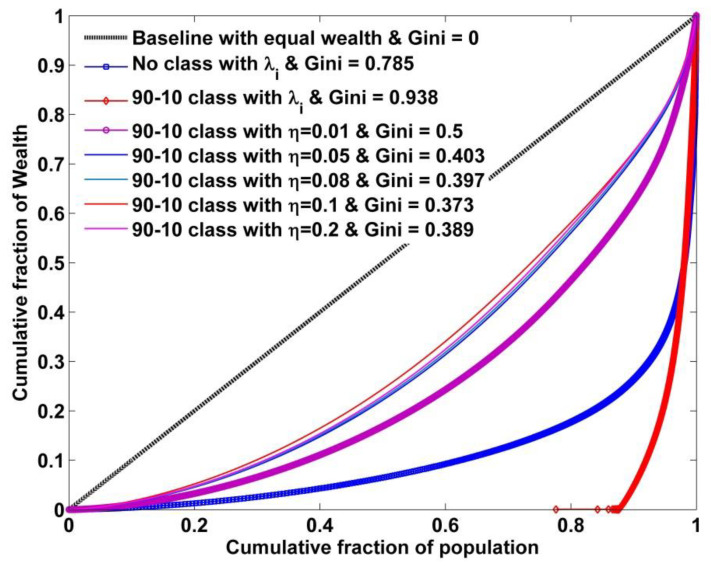
Lorenz curves plotted for the kinetic exchange model with heterogeneous savings under a variety of conditions. Two intriguing behaviors are observed: one is the unilateral flow of wealth by the economic barrier for ta wo-class society and the other is the nonlinear behavior of a solidarity parameter on mitigating wealth inequality. At η=0.1, the Gini coefficient seems to be minimized although numerically.

In order to quantify the overall wealth inequality for the kinetic exchange models considered, we use the Lorenz curve and the Gini coefficient as shown in Figure 7. The Lorenz curve is a standard way of representing income/wealth distribution in the economic literature [36]. It is defined by two coordinates X and Y depending on a wealth parameter x and a wealth density function f(x),

(18) $$X\left(x\right)=\int_{0}^{x}f\left(x^{\prime }\right)dx^{\prime }\;\mathrm{and}\;Y\left(x\right)=\int_{0}^{x}x^{\prime }f\left(x^{\prime }\right)dx^{\prime }/\int_{0}^{\infty }x^{\prime }f\left(x^{\prime }\right)dx^{\prime }$$

where X denotes the fraction of the population with wealth below x, and Y is the fraction of the wealth this population accounts for. As x varies from 0 to ∞, X and Y change from 0 to 1 and parametrically define a curve. We present the Lorenz curves for the kinetic models with heterogeneous savings in Figure 7. Two interesting behaviors are observed: one is the extreme wealth polarization by two-class society with wealth-dependent trading rules, which leads to a unilateral flow of wealth from the poor to the rich, and the other is the nonlinear behavior of solidarity parameters on mitigating social inequality.

In addition, we compute the Shannon entropy [37] to quantify randomness and stochasticity in wealth distributions. Given the numerical histogram, the Shannon entropy is expressed as

(19) $$S\left(w\right)=-\sum_{i=1}^{N}p\left(w_{i}\right)\times \ln p\left(w_{i}\right)$$

where $p\left(w_{i}\right)$ denotes a discrete probability of one’s wealth belonging to $\left[w_{i}-\frac{\Delta w}{2},\;w_{i}+\frac{\Delta w}{2}\right]$ with a bin size Δw. Table 2 contains the computed results of the Shannon entropy and the Gini index. As shown in Table 2, the robustness of the Shannon entropy implies that microscopic replacements among agents make no impact on the stochastic structure of the whole stationary system.

## 4. Discussion and Conclusions

In this study, we have presented a modified agent-based stochastic model by introducing a two-class society with wealth-dependent trading rules and a solidarity formation, in order to explain the dynamic aspects of the Pareto regime and the polymodal behavior of income/wealth distributions observed in some cases [27,28]. According to previous kinetic exchange models, all agents trade in the same manner irrespective of their own wealth. For example, this behavior of random exchange between the poorest and the superrich is in some respects very unreasonable. So we first established a two-class society, which is based on empirical facts [26], and modified the random exchange rule into the wealth-dependent trading rule. In our study, we adopted the 80–20 and 90–10 classifying criteria as rule-of-thumb in order to establish a two-class society. In the forthcoming research, we will closely examine the effect of a classifying criterion. When we applied this modification to the existing kinetic models, a drastic bimodal distribution appeared—that is, there was no intermediate regime in the distribution as shown in Figure 3. This looks very unreal in terms of studies empirically reported so far. Therefore, we incorporated the solidarity-forming factor in our two-class model. This modification is motivated from activities of labor unions and the government’s inequality-mitigating policies. We mainly investigated the impact of our two constraints or modifications on the kinetic exchange model with quenched heterogeneous saving propensity, which yields to a power law distribution for the high end although the Pareto exponent is fixed to one. Using our final stochastic model, we obtained two interesting facts. One is the varying Pareto exponent over varying solidarity parameter, which explains the dynamic aspects of the high end. There is also a varying scale regime as shown in Figure 6. As the solidarity parameter increases, the scale range gets smaller and the Pareto exponent increases. Furthermore, it is noticeable that the Pareto exponent of a two-class society with no solidarity is still estimated to be one as shown in Figure 6. That is, the solidarity parameter is the key ingredient in varying the power-law exponent in income/wealth distribution. The other is the peak-like polymodal behavior observed in Figure 5. In fact, the bimodal and the polymodal income/wealth distributions are not clear in empirical income/wealth distributions so far except those in J.C. Ferrero’s works [27,28], where the Japanese income distribution in 1998 shows an unclear bimodal behavior [27] and the Argentine income distribution shows a two-humped bimodal behavior never observed in other empirical works [28]. This polymodal behavior was not generated from the previous kinetic exchange model. Although our peaked distribution is not the same as those in Japan 1998 and Argentina 2002, our two-class society model with solidarity presents a possibility to generate unfamiliar income distribution rarely observed in reality. 

We give a summary of our numerical results in the following. Our model led an egalitarian society into extreme social polarization with the Gini coefficient estimated to be more than 0.94 and the Shannon entropy to be 0.96 for the case with heterogeneous saving propensity under the condition of no solidarity. Also, the intermediate part of wealth distribution is completely collapsed irrespective of model details considered here. Nevertheless, the high end showed a robust Pareto-law behavior with the same Pareto exponent. Another intriguing finding is that a most even society, that is, a society with higher homogeneous saving propensity is most severely polarized by the economic barrier. This is probably due to the definition of $△x=\varepsilon \times \min\left(x_{i}\left(t\right),x_{j}\left(t\right)\right)$, according to which the △x is greater compared to other cases since the difference between randomly chosen two wealth is smaller as the society gets more and more even. When the solidarity is involved in the two-class society model, a varying Pareto exponent and a poly-model behavior in distribution emerged. This finding can give a clue to understand the varying Pareto exponent in the high end and the polymodal behavior in the intermediate regime in terms of solidarity factors. The solidarity factor can be extended further over our simple argument, that is, the activity of labor unions and the government’s counter-inequality policies. 

Although our model partly succeeded in reproducing the varying Pareto exponent and the polymodal behavior in distributions, there are some critical limitations. First, we just consider the closed economy, which is numerically and analytically well analyzed in terms of kinetic exchange models. In forthcoming research, we will examine the validity of our model in the open economy. Second, a two-class society hypothesis is not universal but in some respects ad-hoc. Therefore, we need to study the possibility of multi-class society. Third, the wealth-dependent trading rules are also ad-hoc, so we need to consider general cases. Lastly, we cautiously propose the possibility of evaluating the government’s counter-inequality policies using our model. 

## Figures and Tables

**Table 1:** Two class-threshold criteria are given for three representative kinetic models. All threshold values are numerically determined from simulated distributions of those models. The distributions are obtained from several hundred steady-state ensembles, each of which is performed from the initial condition of egalitarian society consisting of 1000 agents with the wealth of 100.

Model.	Saving Parameter (λ)	$x_{th}\;\mathrm{Based}\;\mathrm{on}\;80–20$	$x_{th}\;\mathrm{Based}\;\mathrm{on}\;90–10$
**2-1 model**, Equation (4)	No saving	160	230
**2-2 model**, Equation (6)	λ=0.1	60	128
	λ=0.2	156	215
	λ=0.3	152	201
	λ=0.5	147	189
	λ=0.7	138	167
	λ=0.8	128	147
	λ=0.9	122	136
	λ=0.95	115	124
**2-3 model**, Equation (12)	$\lambda_{i}\in U\left(0,1\right)$	110	117

**Table 2:** A kinetic exchange model with quenched heterogeneous savings is only considered because it is most suitable for the real wealth distribution. The entropy is computed numerically with Δw=1 for qualitative comparison. The entropy measure is robust compared to the Gini index over varying solidarity parameter. Also, the Gini index becomes stable for increasing solidarity parameter above a certain value approximately to be =0.03 while the Shannon entropy is robust over varying solidarity parameter at statistical stationary states.

Model	Solidarity Parameter	Entropy, S(w)	Gini Index
**2-5 model**	η=0	0.9595	0.9371
	η=0.01	5.2524	0.4768
	η=0.03	5.2087	0.3874
	η=0.05	5.1369	0.3794
	η=0.08	5.1992	0.3878
	η=0.1	5.1594	0.3728
	η=0.15	5.1608	0.3662
	η=0.2	5.1512	0.3704
